# Supplementary material for: TALEN outperforms Cas9 in editing heterochromatin target sites
Source: Nat Commun. 2021 Jan 27;12:606. doi: 10.1038/s41467-020-20672-5 (PMC7840734; doi:10.1038/s41467-020-20672-5)
Supplement: Supplementary file 1 — Supplementary Information [file 41467_2020_20672_MOESM1_ESM.pdf]

Supplementary Information

TALEN outperforms Cas9 in editing heterochromatin target sites

Surbhi Jain[1†], Saurabh Shukla[2,3†], Che Yang[1], Meng Zhang[2], Zia Fatma[2,4], Manasi Lingamaneni[1], Shireen Abesteh[1], Stephan Thomas Lane[4], Xiong Xiong[2], Yuchuan Wang[5], Charles M. Schroeder[2,6,7], Paul R. Selvin[3,7,8], Huimin Zhao[1,2,4,7*]

[1]Department of Biochemistry, School of Molecular and Cellular Biology, University of Illinois at Urbana−Champaign, Urbana, IL
[2]Department of Chemical and Biomolecular Engineering, University of Illinois at Urbana−Champaign, Urbana, IL
[3]Center for the Physics of Living Cells, University of Illinois at Urbana−Champaign, Urbana, IL
[4]Carl R. Woese Institute for Genomic Biology, University of Illinois at Urbana−Champaign, Urbana, IL
[5]Computational Biology Department, School of Computer Science, Carnegie Mellon University, Pittsburgh, PA 15213, USA
[6]Beckman Institute for Advanced Science and Technology, University of Illinois at Urbana-Champaign, Urbana, IL
[7]Center for Biophysics and Quantitative Biology, University of Illinois at Urbana−Champaign, Urbana, IL
[8]Department of Physics, University of Illinois at Urbana−Champaign, Urbana, IL

[†]These authors contributed equally to this work.

This PDF file includes:
Supplementary Figures 1-9
Supplementary Tables 1-5

Supplementary figures

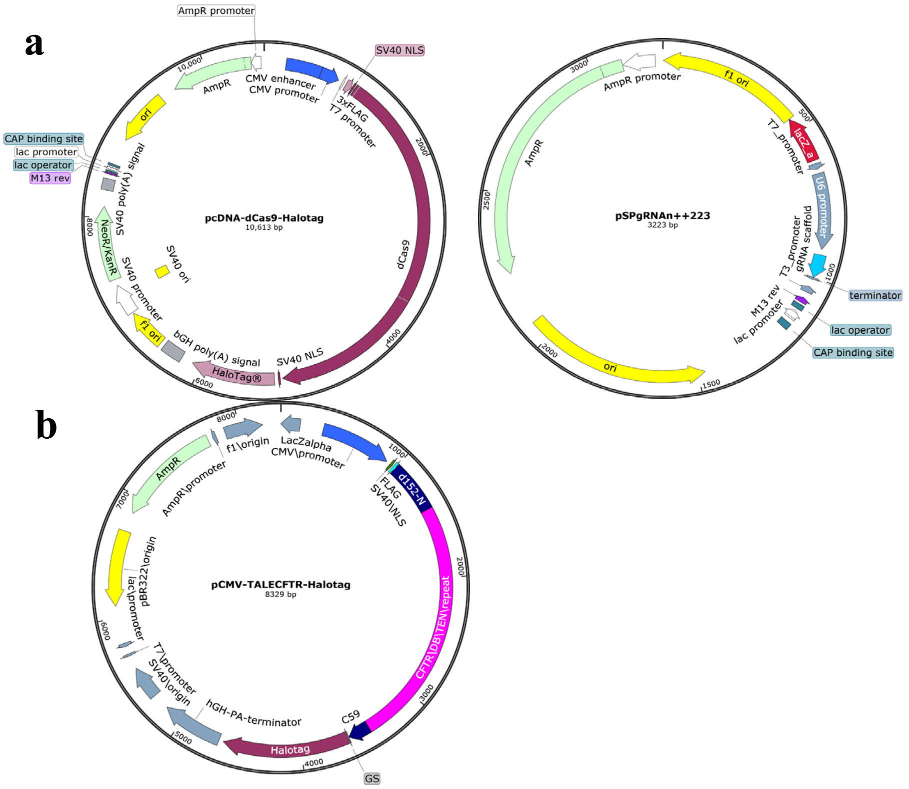

Supplementary Fig. 1. Components of DBD-Halotag plasmid backbones. **a** dCas9 protein variants were constructed by cloning different 20 bp gRNA in pSPgRNA plasmid backbone. **b** TALE variants were assembled specifically for each target in a plasmid backbone with optimized N- and C-terminal domains. Halotag is genetically fused to dCas9 and TALE at the C-terminal.

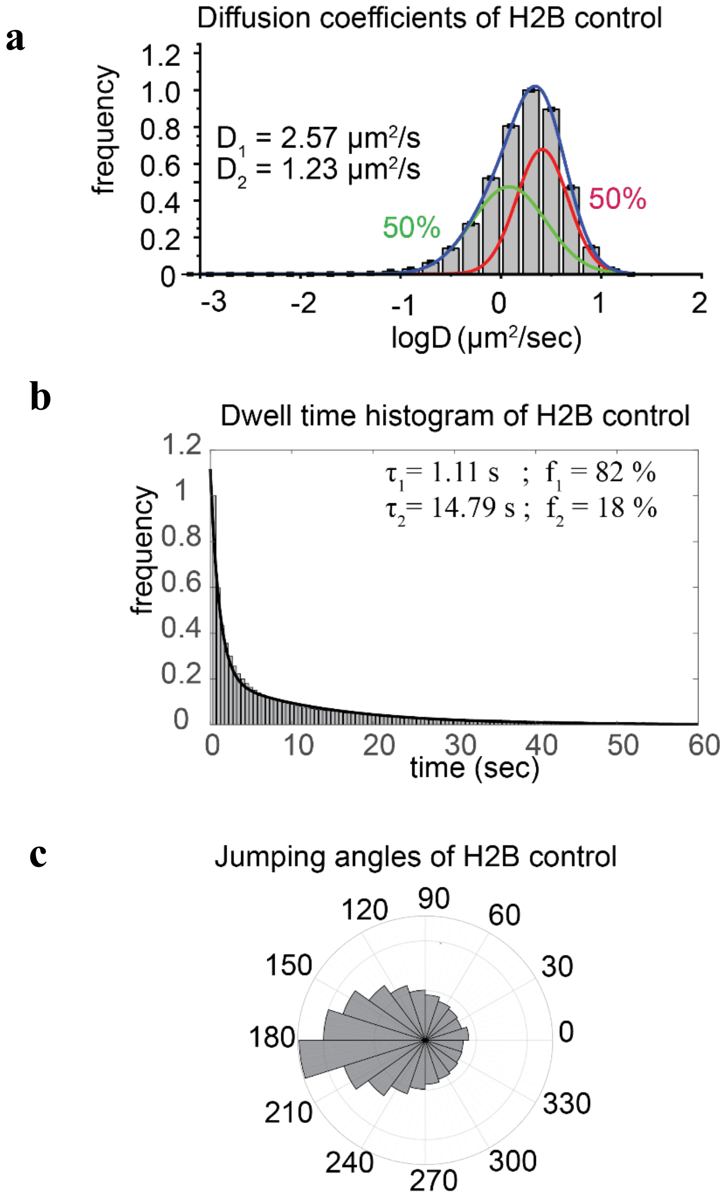

Supplementary Fig. 2. Diffusion characteristics of H2B control. a Diffusion histogram of H2B is also fitted by two peaks that indicate characteristic two modes of diffusion. **b** In long exposure time condition (500 ms), we observe bound H2B molecules. The residence time histogram of H2B is best described by a two-component exponential decay model with a longer binding time of 14.79 seconds and a shorter binding time of 1.11 s. **c** Jumping angles distribution of H2B is highly skewed towards 180º C that characterizes highly constricted motion in the genome. Because of the overexpression of H2B molecules, unbound H2B proteins are captured with our short imaging time condition. Therefore, we observe a relatively smaller number of bound H2B condition in this imaging condition that is more prominently observed in the jumping angle plot. The skewness factor for H2B is 2.61. Source data are provided as a Source Data file.

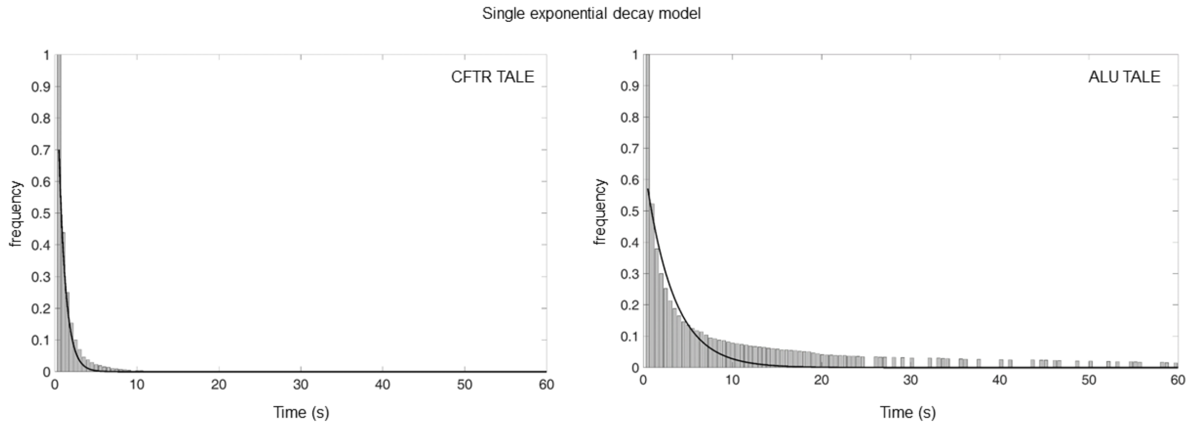

Supplementary Fig. 3. Single exponential decay model fit. The residence time distribution of CFTR and Alu TALE is fitted with a single component exponential decay model. The model does not fit the data for both CFTR and Alu-TALE. Source data are provided as a Source Data file.

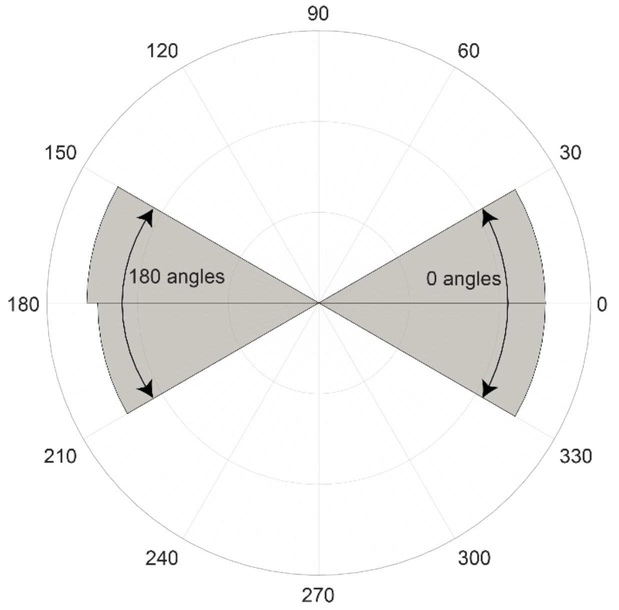

$$\text{Skewness factor} = \frac{\text{0 angles}}{\text{180 angles}}$$

Supplementary Fig. 4. Skewness factor for jumping angle distributions. Skewness factor is defined to quantify the asymmetry of jumping angles of the proteins. Skewness factor is defined as the ratio of '0 angles' and '180 angles'. '0 angles' are the angles that are biased towards 0º (between -330 º to 30 º). '180 angles' are the angles that are biased towards 180 º (angles between 150 º to 210 º).

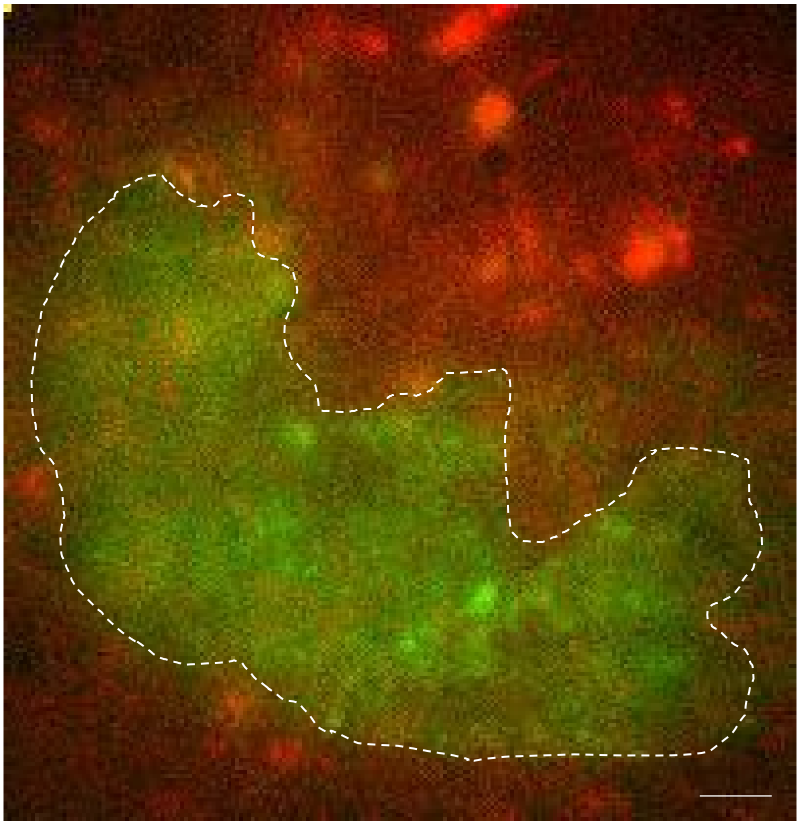

Supplementary Fig. 5. **Snapshot from a movie of GFP heterochromatin mask (dotted area).** HeLa cell stably expressing GFP-HP1a protein. HP1a is known to extensively associate and stabilize heterochromatin. Heterochromatin acts as a barrier for searching gene-editing proteins. Red false-color represents Alu TALE molecules, and green false-color represents the heterochromatin region. Scale bar: 0.1 µm

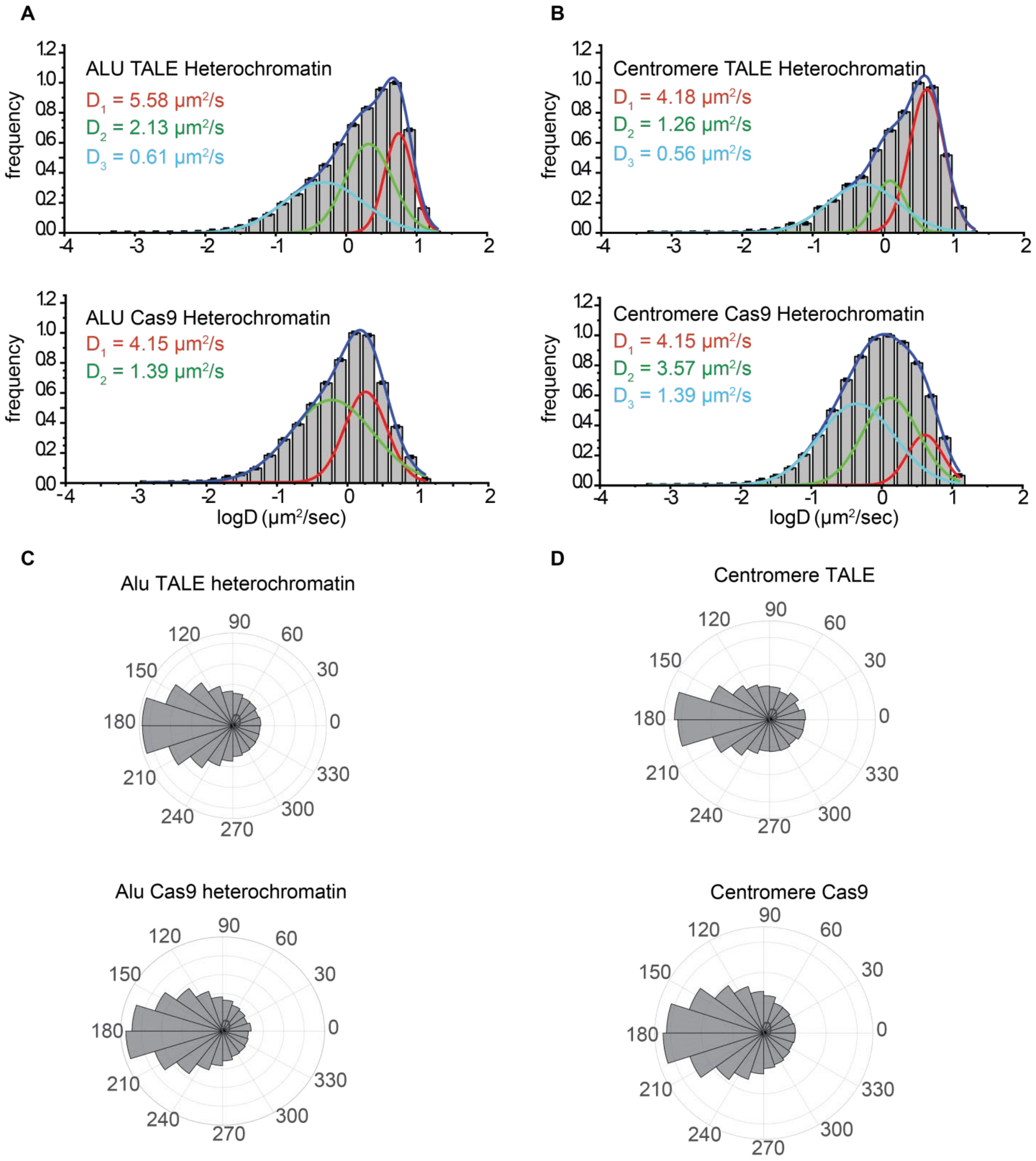

Supplementary Fig. 6. Chromatin context-dependent search of TALE and dCas9 in heterochromatin. a Histograms of diffusion coefficients of Alu-TALE and Alu-dCas9 in heterochromatin region are plotted. ALU-TALE has three distinctly diffusing populations in the heterochromatin region as compared to Cas9, that has two subpopulations. **b** In the centromere region, both TALE and dCas9 have three distinctly diffusing populations. However, the profile of D histograms, i.e., the proportion of each population, is different in TALE and dCas9. The intermediate population appears to correspond to hopping behavior as in the case of dCas9; it only appears for centromeres, which is a tightly packed heterochromatin feature consisting of tandem repetitive sequences. TALE local search process is non-specific and seems to be affected by a high

concentration of DNA in a tightly packed space, so we observe hopping behavior for centromere as well as Alu retrotransposon heterochromatin features that we study. **c,** and **d,** Both TALE and dCas9 are highly skewed towards -180º in the heterochromatin region. The degree of skewness varies as the target sequence is varied for both dCas9 and TALE. TALE and dCas9 molecules are experiencing a densely packed nuclear environment. Alu and Centromere targeting TALEs and dCas9 variants are used to characterize the search processes in prominent heterochromatin structural elements of a mammalian genome. In the case of centromeric structures, the target sites are highly repetitive and concentrated, and we observe a 'hopping' like the behavior of TALE and dCas9 proteins, as shown in Extended Fig. 5. We further show that this hopping behavior depends on the presence of similar sites in close proximity for target-searching dCas9 molecules. dCas9 targeting Alu retrotransposon elements, which are not concentrated but are interspersed throughout the genome, do not exhibit hopping behavior, which suggests that the target search process of these proteins in heterochromatin is fundamentally different. For TALEs, the hopping behavior is seemingly dependent on the compaction of the chromatin, but for dCas9, there is an additional requirement, perhaps the increased concentration of PAM sites or a seed-region including PAM-site. Source data are provided as a Source Data file.

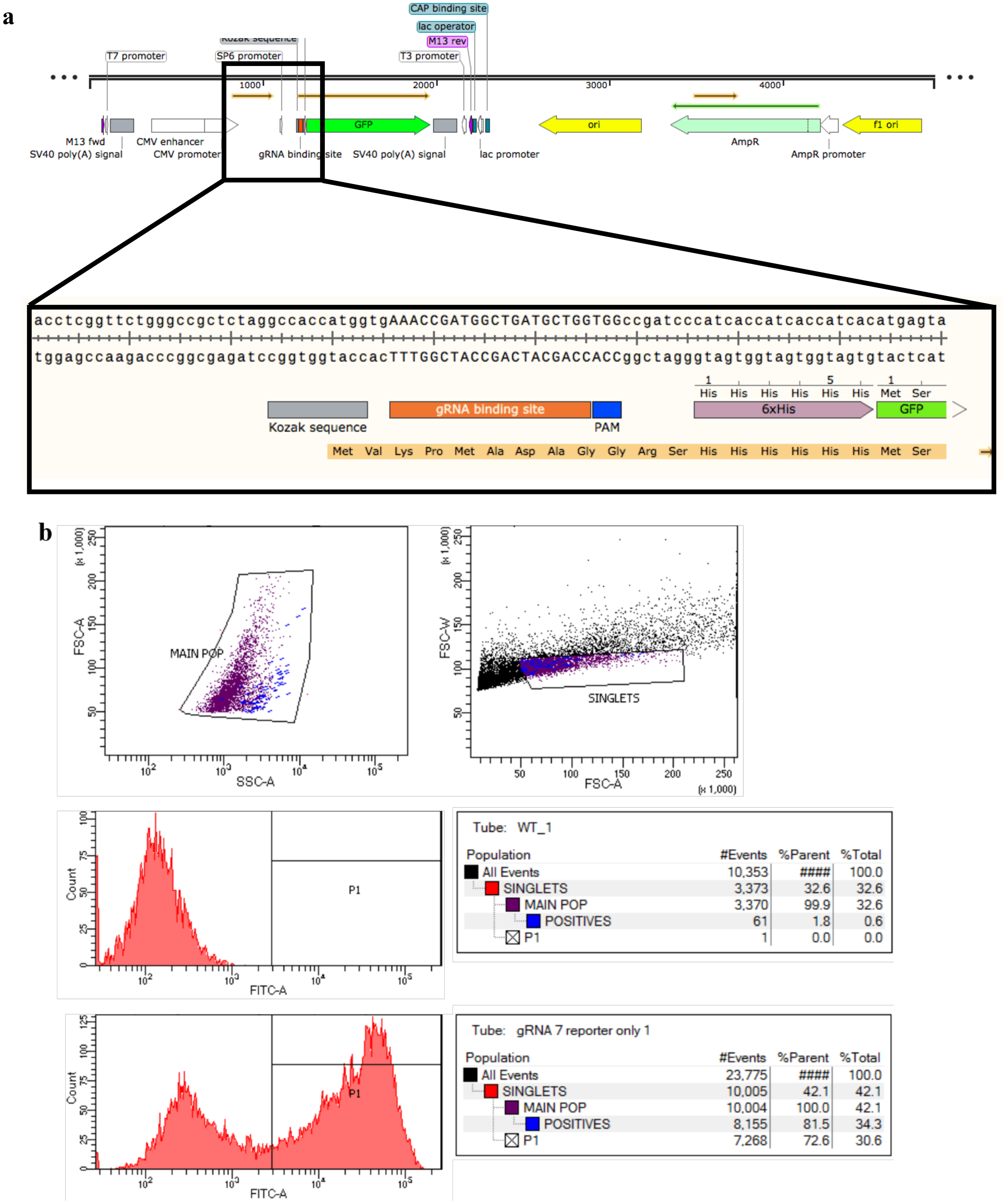

Supplementary Fig. 7. a Design of the reporter assay includes cloning sites for gRNA binding site, including PAM at the 5'-end of GFP start codon. Successful editing events will result in loss of fluorescence as measured by Flow cytometry. No change in fluorescence refers to the inactivity of gRNA construct. **b** Gating strategy to determine population characteristics of GFP+ cells. P1 is gated based on WT non-fluorescent cells and represents GFP+ cell population.

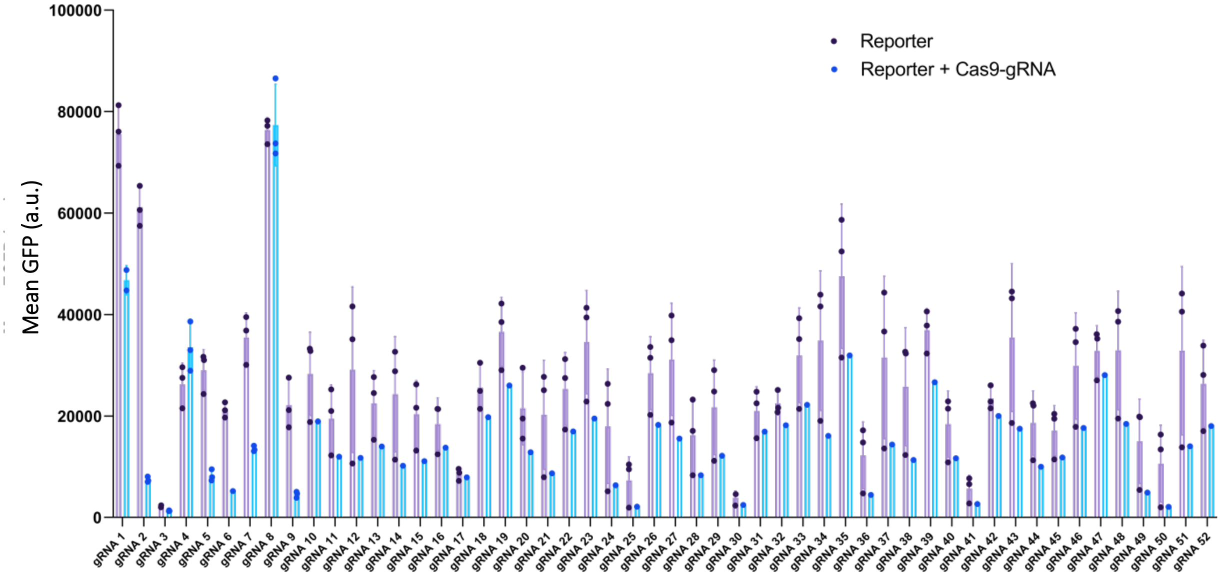

gRNA 1 ACTIVE	gRNA 2 ACTIVE	gRNA 3 ACTIVE	gRNA 4 INACTIVE	gRNA 5 ACTIVE	gRNA 6 ACTIVE	gRNA 7 ACTIVE	gRNA 8 INACTIVE
gRNA 9 ACTIVE	gRNA 10 ACTIVE	gRNA 11 ACTIVE	gRNA 12 ACTIVE	gRNA 13 ACTIVE	gRNA 14 ACTIVE	gRNA 15 ACTIVE	gRNA 16 ACTIVE
gRNA 17 INACTIVE	gRNA 18 INACTIVE	gRNA 19 ACTIVE	gRNA 20 INACTIVE	gRNA 21 ACTIVE	gRNA 22 ACTIVE	gRNA 23 ACTIVE	gRNA 24 ACTIVE
gRNA 25 ACTIVE	gRNA 26 ACTIVE	gRNA 27 ACTIVE	gRNA 28 INACTIVE	gRNA 29 ACTIVE	gRNA 30 ACTIVE	gRNA 31 ACTIVE	gRNA 32 INACTIVE
gRNA33 ACTIVE	gRNA34 ACTIVE	gRNA35 ACTIVE	gRNA36 ACTIVE	gRNA37 ACTIVE	gRNA38 ACTIVE	gRNA39 INACTIVE	gRNA40 ACTIVE
gRNA41 ACTIVE	gRNA42 INACTIVE	gRNA43 ACTIVE	gRNA44 ACTIVE	gRNA45 ACTIVE	gRNA46 ACTIVE	gRNA47 ACTIVE	gRNA48 ACTIVE
gRNA49 ACTIVE	gRNA50 ACTIVE	gRNA51 ACTIVE	gRNA52 ACTIVE				

Supplementary Fig. 8. GFP reporter assay to assess gRNA activity. Y-axis represents the arithmetic mean of GFP fluorescence. Reporter only samples are compared to samples with reporter and Cas9-gRNA by a 2-tailed t-test. n = 3 biological replicates. Data are presented as mean values +/-SEM. 9/52 (17.3%) gRNAs showed no editing activity in the reporter assay and were not used for TIDE analysis. Source data are provided as a Source Data file.

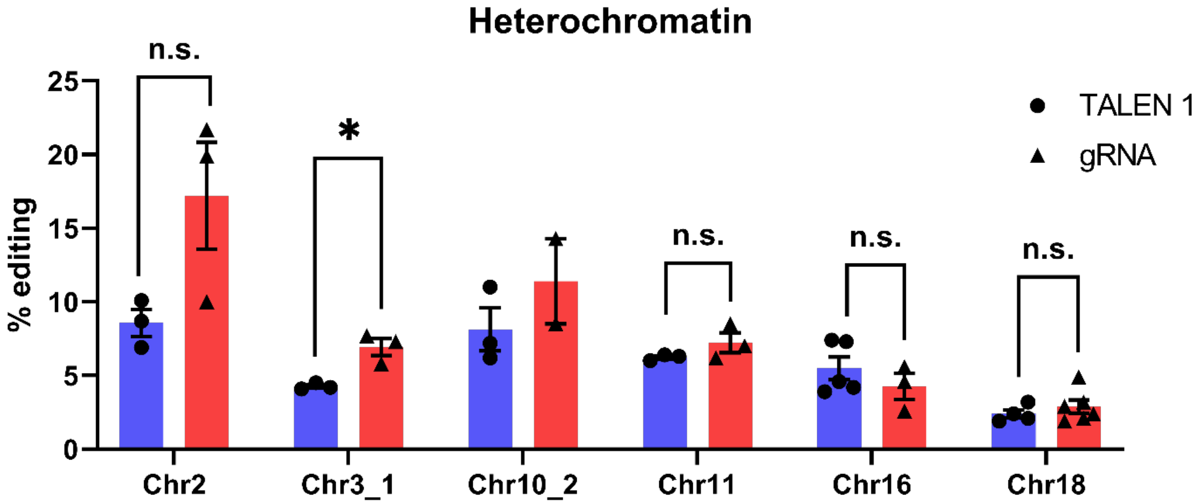

Supplementary Fig. 9. Cas9 and TALEN perform similarly in 5/12 loci (41.66%), and CRISPR performs better than TALEN at 1/12 loci (0.08%). Data are presented as mean values +/-SEM. TALEN samples are compared to Cas9-gRNA samples by a 2-tailed t-test when n>2. Error bars represent the standard error of the mean. $p < 0.05$*, $p < 0.01$**, $p < 0.001$*** **.** p values are given in the bracket: Chr2 (0.0829), Chr3_1 (0.9656), Chr10_2 (0.508), Chr11 (0.217), Chr16 (0.36), Chr18 (0.928). Source data are provided as a Source Data file.

Supplementary Table 1. DNA sequences targeted by TALE and Cas9 variants used in single-molecule imaging.

**Name**	**DNA sequence**
CFTR TALE	CCAGGAAAAAAAGAGGAGT
Alu TALE	CTCGAACTCCTGACCTCAGG
Centromere TALE	CTTTTTGTAGAATCTGCAAG
CFTR gRNA	AGTATGCAAGAGCTACATAA
Alu gRNA	CGGGCGGATCACCTGAGGTC
Centromere gRNA	CTTTTTGTAGAATCTGCAAG
TALEN 16	GCCATTGTGCTATTTGCTCG
gRNA 9	CTATACCTTTACCGATAGCA

Supplementary Table 2. HCT116 chromosome co-ordinates of heterochromatin loci for genome editing protein editing efficiency comparison.

Chromosome co-ordinates	H3K9me3 fold change	H3K27me3 fold change
chr2:29,682,862-29,683,365	3.3248	
chr3:20,900,087-20,900,661		4.12723
chr3:140,209,900-140,210,417	3.63982	
chr4:23,453,929-23,454,428		4.11628
chr10:9,506,171-9,506,675		3.22333
chr11:89,501,656-89,502,155	3.41277	
chr17:5,926,127-5,926,627	2.61907	
chr17:51,406,872-51,407,371	4.67405	
chr16:79,820,420-79,820,918	6.98685	
chr7:103,199,804-103,200,302		4.0682
chr10:7,745,157-7,745,658		2.54284
chr18:44,552,052-44,552,552		9.54758

Supplementary Table 3. List of gRNAs designed for TIDE analysis using CHOPCHOP and Benchling.

CHOPCHOP

Heterochromatin region	Target sequence	Genomic location	Strand	Efficiency
chr17:51,406,872-51,407,371				
1	ATATAATGCTGACCGAATTCAGG	chr17:51407051	-	24.34
2	GGACCATATAGGCAGTGAAAAGG	chr17:51407114	-	46.81
chr18:44,552,052-44,552,552				
3	TCCCGCTCGAAAACACAGAAAGG	chr16:79820686	-	66.51
4	TAGCTCATCGATGCCCCCACTGG	chr16:79820461	-	57.08
chr7:103,199,804-103,200,302				
5	GATGCATGGGTTAGGTATCATGG	chr7:103199932	-	41.85
6	ATAGATTCATACTTCAGGACAGG	chr7:103199835	+	52.91
chr10:7,745,157-7,745,658				
7	CTATTGGAATCGCTATTCAATGG	chr10:7745539	+	46.93
8	TACAGATCACTCCTTGCTGTTGG	chr10:7745491	+	53.48
chr16:79,820,420-79,820,918				
9	GGTTATAATAGGTGTGACAATGG	chr18:44552243	-	61.86
10	GCTAGTGATGTTAATAGTAGTGG	chr18:44552359	-	54.08
chr2:29,682,862-29,683,365				
	ATATGACTTATGTCCTCCGCTGG	chr2:29683223	-	49.23
	TATGACTTATGTCCTCCGCTGGG	chr2:29683222	-	62.67
chr3:20,900,087-20,900,661				
	GCTGCTTAATAACATTAGGCAGG	chr3:20900091	-	54.11
	GCTAGAAAGATGCTTCAGACAGG	chr3:20900296	-	60.75
chr3:140,209,900-140,210,417				
	ACCTTACCACCAGCAAATCCAGG	chr3:140210124	-	50.01
	AGGCCGGAGGGGCACCAGACTGG	chr3:140210006	-	40.3
chr4:23,453,929-23,454,428				
	AAGTAATCCGAGACAATTAGAGG	chr4:23454140	-	61.38
	GTTGTCCACCGAGTCTACCTGGG	chr4:23454351	+	55.84
chr10:9,506,171-9,506,675				
	AGTCTATATGACACTATGATGGG	chr10:9506421	+	57.55
	AAACAAACGGGCCATTCTGCTGG	chr10:9506562	+	38.34
chr11:89,501,656-89,502,155				

	TAATGGCCATCAATGCAAGCTGG	chr11:89501953	-	56.49
	TAATGTCTGATAAGAGGCATAGG	chr11:89501793	+	55.2
chr17:5,926,127-5,926,627				
	TATACAGGGCCTTTTAGGGGTGG	chr17:5926281	-	58.73
	CTTCACGGCTTTTGTTCTAAGGG	chr17:5926362	-	43.17

Benchling

**Heterochromatin region**	**Position**	**Strand**	**Sequence**	**PAM**	**On-Target Score**	**Off-Target Score**
chr17:51,406,872-51,407,371	51406947	-	gaaaatggtaatcagcatgg	agg	74.1	56.6
	51406914	-	cacaggctcagccttaacaa	agg	69.1	53.7
chr16:79,820,420-79,820,918						
	79820818	-	gctggtagtagtgataatgg	tgg	80.8	32.1
	79820560	-	aatgatgatagtgagcatga	tgg	72.3	50.2
chr7:103,199,804-103,200,302						
	103200178	-	caacacttagccaggcacag	tgg	73.6	48.1
	103200065	+	agtcttgctctgttgcccag	tgg	73	29.2
chr10:7,745,157-7,745,658						
	7745185	-	tgggtggatcgaacacccag	agg	75.9	76.7
	7745508	-	tcatgaacaagccaacagca	agg	67.2	57
chr18:44,552,052-44,552,552						
	44552345	-	tggcatgctactgaaaacag	cgg	83.9	32.1
	44552417	-	catctcaaaacacaaacaca	agg	69.4	28
chr2:29,682,862-29,683,365						
	29682945	-	tcactaaaacctcttcactg	tgg	73.6	57.8
	29683224	+	taagcacatttgttacccag	cgg	70.5	61.3
chr2:78,431,559-78,432,060						
	78431942	+	ctataagatgaacgtcacca	agg	74.3	84.6
	78431967	+	tatactcaccagactaccca	agg	68.2	74.8
chr3:20,900,087-20,900,661						
	20900115	+	aatgttattaagcagccaaa	ggg	61	35.5
	20900302	-	gctagaaagatgcttcagac	agg	60.7	68.7
chr3:140,209,900-140,210,417						

	140210058	-	ggtttttagaaagatcatga	agg	64.8	48.5
	140210113	-	tccagggcaaggatgaaatg	agg	63.2	48.8
chr4:23,453,929-23,454,428						
	23454065	-	aataataatagtaaagtaca	ggg	70.4	30.5
	23454315	-	agagttgcgggagaccaagg	tgg	68.9	60.1
chr8:7,351,500-7,352,011						
	7351686	-	catgatgtaagtcggctgag	ggg	74.2	46.9
	7351794	-	agaaacaatataaatgtctg	ggg	66.7	29.7
chr10:9,506,171-9,506,675						
	9506394	+	gaccaataggcagagcacaa	agg	66.2	63.8
	9506364	+	acattaacaaaaggggttgg	ggg	63.1	52.9
chr11:89,501,656-89,502,155						
	89502129	+	caaggtgggtggatcacctg	agg	64.6	71.7
	89501992	-	ccaggatttgggagacatgg	tgg	64.4	16.6
chr13:56,045,589-56,046,141						
	56045841	-	ggcaaggtgttcatgcaaag	agg	69.5	67
	56045787	+	aagaagacacttgatcaaag	tgg	68	57.5
chr17:5,926,127-5,926,627						
	5926552	+	tgctcctgcaaagatcagtg	tgg	71.3	37.9
	5926330	-	ggggaaactgaggctcaaag	agg	69.4	17.4

Euchromatin gRNAs

Gene	Target site	PAM
EMX1	GAGTCCGAGCAGAAGAAGAA	GGG
TPCN2	GTGGGTGAGTGAGTGCGTGC	GGG
NRG2	GGGCAGTTTGCTCCTGGCAC	AGG
DNMT1	GATTCCTGGTGCCAGAAACA	GGG

Supplementary Table 4. List of TALEN pairs designed for TIDE analysis using CHOPCHOP and SAPTA.

#	Name	TALE Forward Binding	TALE Reverse Binding
1	chr2-CHOPCHOP	tgcaatacttgatcattg	tacccagcggaggacata
2	chr2-SAPTA	tagtgactaccttgcagtag	cagcccccacatcttgct
3	chr3_1-CHOPCHOP	tagtgactgatctgggaa	aaagctcggctagagcta
4	chr3_1-SAPTA	acacaacctataattgat	gtaccattgtaagaacctttct
5	chr3_2-CHOPCHOP	taagtcatacgtccctaa	tatctgtggagtaaaaca
6	chr3_2-SAPTA	ctactttgccttcatgat	gtgtcttagcaaggcag
7	chr4-CHOPCHOP	ttagcctgcgtgagtacc	attgtctcggattactta
8	chr4-SAPTA	ccacccaagccctgtac	atttctagcaaccagat
9	chr7-CHOPCHOP	cggtccatgatacctaa	taagacatgaagaaatg
10	chr7-SAPTA	cagcccccactgtagctgggac	ccaacacttagccaggcacag
11	chr10_1-CHOPCHOP	cttaaagcgaactgtac	cctactgaaccaagttc
12	chr10_1-SAPTA	cctccccagaccatctgct	cttttcattttgctggacag
13	chr10_2-CHOPCHOP	tgcaaggtagtttgatac	taaaaaaacactatatca
14	chr10_2-SAPTA	gaaccctaatttgaacc	gtacattgacacatcattat
15	chr11-CHOPCHOP	tcacagcacgccttgtgc	cattaacataccttgcca
16	chr11-SAPTA	ttgccaccaaatagttgat	gcctctttttaggattcag
17	chr16-CHOPCHOP	tactattgctaccatca	gtggtagtaacggtgat
18	chr16-SAPTA	actaccaccatcaccac	tgctcactggcaccattgtcac
19	chr17_1-CHOPCHOP	tccccatctgtaaataaa	ccgtgaaggtccttccaa
20	chr17_1-SAPTA	gtggcttgcatcctacatggctgag	ctgccagacactgcgttggattgt
21	chr17_2-CHOPCHOP	aatggaatgctgttctc	ataatgctgaccgaatt
22	chr17_2-SAPTA	ttgccttatattcctttg	ggaccatataggcag
23	chr18-CHOPCHOP	gtgcgggggttgtttcc	gctcccacagcgacaat
24	chr18-SAPTA	gcagccgctgttttcagtag	ctagcccctcatagctcatcgat
25	EMX1_CHOPCHOP	tccgaggaaccgctccgg	tcaccccagcgcggccga
26	EMX1_SAPTA	ggttccagaaccggag	caggcccttcctcctccagcttct
27	TPCN2_CHOPCHOP	tgcgctaccgcgctgctc	cctgaccgagagtgtcga
28	TPCN2_SAPTA	ttggcgtctccaggccacag	ctgcctcccaacccactgcag
29	NRG2_CHOPCHOP	tcggccaggcggtaactg	cgacacggcgtgcgccga
30	NRG2_SAPTA	ttgctcctggcacag	gtgcccactgcactgaa
31	DNMT1_CHOPCHOP	tcgatctcttacctcgat	tcataggtcgagtcggaa
32	DNMT1_SAPTA	tagcagcttcctcctcctttat	tccccagagtgacttttccttttat

Supplementary Table 5. List of genotyping primers for TIDE analysis.

**Genotyping primers**	**Sequence**	**TIDE analysis**
chr2-HCT116-F	GCGAATCTCTGTTTAGATCACC	gRNA and TALENs
chr2-HCT116-R	GGTCTGTCTCAGCTTTCAAC	gRNA and TALENs
chr3_1-HCT116-F	GCAGGTCATTTGTGTCACAAG	gRNA and TALENs
chr3_1-HCT116-R	GTGACCCTTTCTTAGGATGTTC	gRNA and TALENs
chr3_2-HCT116-F	CAGCTGTGATGTCTAGTACC	gRNA and TALENs
chr3_2-HCT116-R	GACAGCGTCTCACTCCAG	gRNA and TALENs
chr4-HCT116-F	GCCTTCTAATTTTGATAGGATGC	gRNA and TALENs
chr4-HCT116-R	GATGCTGTTATCTGCAAAGG	gRNA and TALENs
chr7-HCT116-F	GTGGTGATGGTAGGATTTGAAC	gRNA and TALENs
chr7-HCT116-R	GATTACAAATCATGCTCACTTTC	gRNA and TALENs
chr10_1-HCT116-F	ACATTCAAGCACATCCATCTGC	gRNA and TALENs
chr10_1-HCT116-R	CTGCCTGCTATCTTCCGCTT	gRNA and TALENs
chr10_2-HCT116-F	AGCAAACAAGAACTAGAGG	gRNA and TALENs
chr10_2-HCT116-R	TTTCCTCACATCTGCTGTG	gRNA and TALENs
chr11-HCT116-F	CTCACTCCTGCAAGGAAGTAG	gRNA and TALENs
chr11-HCT116-R	CTGCAGGAAGATCTAGAGCTAC	gRNA and TALENs
chr16-HCT116-F	TCAAGGGCAGTGAATGAGAG	gRNA and TALENs
chr16-HCT116-R	AGTCCACGAAAATAGCAGCC	gRNA and TALENs
chr17_1-HCT116-F	CGATGTTAAAGGAAATAGCTC	gRNA and TALENs
chr17_1-HCT116-R	TGAGAGTTTCAGGATCAAGG	gRNA and TALENs
chr17_2-HCT116-F	ATAGCAGAAGCAGCTGGAAG	gRNA and TALENs
chr17_2-HCT116-R	GTCAACGTCCTGTCTTGGATC	gRNA and TALENs
chr18-HCT116-F	AGAATTACCCTCCGTGTTGTG	gRNA and TALENs
chr18-HCT116-R	CCGTCTCTTCTAAAAAGACG	gRNA and TALENs
EMX1-GS-F	CTAGGATGCACAGCAGCTC	gRNA and SAPTA TALEN
EMX1-GS-R	TGGAGGTAGAGACCAGGGTC	gRNA and SAPTA TALEN
TPCN2-GS-F	CTCAGTGCTATCCACAGGTTC	gRNA and SAPTA TALEN
TPCN2-GS-R	GAGCTCCCTGCTGTACAAAG	gRNA and SAPTA TALEN
DNMT1-GS-F	CAGAATGCACAAAGTACTGC	gRNA and SAPTA TALEN
DNMT1-GS-R	AGGTTGTCCTCCATCTGAG	gRNA and SAPTA TALEN
EMX1-TC-F	TGTGCATGTGCCTGGCTG	CHOPCHOP TALEN
EMX1-TC-R	AGAGAATTGGGCAGGCTGTG	CHOPCHOP TALEN
TPCN2-TC-F	GGCAATGGAGCTTTGAGCAG	CHOPCHOP TALEN
TPCN2-TC-R	CTCTCCTCACAGCAGCACTG	CHOPCHOP TALEN
DNMT1-TC-F	GTCATAACTCTCCACCTGCTC	CHOPCHOP TALEN
DNMT1-TC-R	CAGCTACTTGGGAGGCTATG	CHOPCHOP TALEN
