# Supplementary material for: TALEN outperforms Cas9 in editing heterochromatin target sites
Source: Nat Commun. 2021 Jan 27;12:606. doi: 10.1038/s41467-020-20672-5 (PMC7840734; doi:10.1038/s41467-020-20672-5)
Supplement: Supplementary file 3 — Description of Additional Supplementary Files [file 41467_2020_20672_MOESM3_ESM.pdf]

Description of Additional Supplementary Files

File Name: Supplementary Movie 1
Description: CFTR TALE (red dots) fused with JF549 dye moving in a live HeLa cell
