# Supplementary material for: TALEN outperforms Cas9 in editing heterochromatin target sites
Source: Nat Commun. 2021 Jan 27;12:606. doi: 10.1038/s41467-020-20672-5 (PMC7840734; doi:10.1038/s41467-020-20672-5)
Supplement: Supplementary file 5 — Reporting Summary [file 41467_2020_20672_MOESM5_ESM.pdf]

nature research

Corresponding author(s): Huimin Zhao

Last updated by author(s): Nov 16, 2020

Reporting Summary

Nature Research wishes to improve the reproducibility of the work that we publish. This form provides structure for consistency and transparency in reporting. For further information on Nature Research policies, see our Editorial Policies and the Editorial Policy Checklist.

Statistics

For all statistical analyses, confirm that the following items are present in the figure legend, table legend, main text, or Methods section.

n/a	Confirmed	
☐	☒	The exact sample size (*n*) for each experimental group/condition, given as a discrete number and unit of measurement
☐	☒	A statement on whether measurements were taken from distinct samples or whether the same sample was measured repeatedly
☐	☒	The statistical test(s) used AND whether they are one- or two-sided<br>*Only common tests should be described solely by name; describe more complex techniques in the Methods section.*
☒	☐	A description of all covariates tested
☐	☒	A description of any assumptions or corrections, such as tests of normality and adjustment for multiple comparisons
☐	☒	A full description of the statistical parameters including central tendency (e.g. means) or other basic estimates (e.g. regression coefficient) AND variation (e.g. standard deviation) or associated estimates of uncertainty (e.g. confidence intervals)
☐	☒	For null hypothesis testing, the test statistic (e.g. *F*, *t*, *r*) with confidence intervals, effect sizes, degrees of freedom and *P* value noted<br>*Give P values as exact values whenever suitable.*
☒	☐	For Bayesian analysis, information on the choice of priors and Markov chain Monte Carlo settings
☒	☐	For hierarchical and complex designs, identification of the appropriate level for tests and full reporting of outcomes
☒	☐	Estimates of effect sizes (e.g. Cohen's *d*, Pearson's *r*), indicating how they were calculated

Our web collection on statistics for biologists contains articles on many of the points above.

Software and code

Policy information about availability of computer code

Data collection	Nikon NIS Element
Data analysis	Fiji, MATLAB 2016, TrackMate, TIDE version 2.0.1, GitHub link for custom codes (https://github.com/sshukla101/SelvinLab/blob/master/Diffusion_Coefficient_Calculation_CVE_Estimator.m)

For manuscripts utilizing custom algorithms or software that are central to the research but not yet described in published literature, software must be made available to editors and reviewers. We strongly encourage code deposition in a community repository (e.g. GitHub). See the Nature Research guidelines for submitting code & software for further information.

Data

Policy information about availability of data

All manuscripts must include a data availability statement. This statement should provide the following information, where applicable:

- Accession codes, unique identifiers, or web links for publicly available datasets
- A list of figures that have associated raw data
- A description of any restrictions on data availability

All data is available in the main text or the supplementary information text and files. Source data are provided with this paper. Genomic loci sequence files are provided in the supplementary data file. Single molecule imaging raw datasets are available from the corresponding author upon request.

Field-specific reporting

Please select the one below that is the best fit for your research. If you are not sure, read the appropriate sections before making your selection.

☒ Life sciences ☐ Behavioural & social sciences ☐ Ecological, evolutionary & environmental sciences

For a reference copy of the document with all sections, see nature.com/documents/nr-reporting-summary-flat.pdf

Life sciences study design

All studies must disclose on these points even when the disclosure is negative.

Sample size	No sample size calculation was applied in this study to predetermine sample sizes for single-molecule experiments as well as experiments using cell lines. A sample size of three was used as to evaluate the spread of the data for experiments using cell lines and was determined based upon other studies with similar methodologies (DOI: 10.1038/nbt.3290, 10.1016/j.jgg.2017.03.004).
Data exclusions	No data was excluded.
Replication	Single molecule imaging was performed on at least 2 different days with biological replicates and TIDE analysis was replicated twice for first batch of genomic loci. TIDE analysis was not replicated for second batch of TIDE analysis as we doubled the number of constructs tested compared to first batch.
Randomization	Cells were randomly chosen for imaging and subsequent quantification on the experiment (single cell fluorescence microscopy). For other experiments samples were randomly chosen and cells were analyzed in bulk.
Blinding	No blinding was performed for single molecule analysis as well as molecular biology assays as same identity of the samples was part of the data collection and analysis as is standard for similar studies. (DOI:10.1126/science.aac6572)

Reporting for specific materials, systems and methods

We require information from authors about some types of materials, experimental systems and methods used in many studies. Here, indicate whether each material, system or method listed is relevant to your study. If you are not sure if a list item applies to your research, read the appropriate section before selecting a response.

Materials & experimental systems

n/a	Involved in the study	
☒	☐	Antibodies
☐	☒	Eukaryotic cell lines
☒	☐	Palaeontology and archaeology
☒	☐	Animals and other organisms
☒	☐	Human research participants
☒	☐	Clinical data
☒	☐	Dual use research of concern

Methods

n/a	Involved in the study	
☒	☐	ChIP-seq
☐	☒	Flow cytometry
☒	☐	MRI-based neuroimaging

Eukaryotic cell lines

Policy information about cell lines

Cell line source(s)	HeLa (ATCC® CCL-2™), HCT 116 (ATCC® CCL-247™), HEK293T (ATCC® CRL-3216™)
Authentication	None of the cell lines were authenticated
Mycoplasma contamination	The cell lines were not tested for mycoplasma contamination
Commonly misidentified lines (See ICLAC register)	We did not use any commonly misidentified cell lines

Flow Cytometry

Plots

Confirm that:

☒ The axis labels state the marker and fluorochrome used (e.g. CD4-FITC).

☒ The axis scales are clearly visible. Include numbers along axes only for bottom left plot of group (a 'group' is an analysis of identical markers).

☒ All plots are contour plots with outliers or pseudocolor plots.

☒ A numerical value for number of cells or percentage (with statistics) is provided.

Methodology

Sample preparation	Cells were detached from culturing plates and resuspended in 0.5mL phosphate buffered saline.
Instrument	BD LSR II
Software	FCS Express 6
Cell population abundance	HCT116 cells were analyzed to determine mean GFP fluorescence compared to WT (non-GFP fluorescent cells). Cell populations were not sorted.
Gating strategy	Cell debris were excluded by gating for main population in SSC-A/FSC-A. Singlets were gated for using FSC-A/FSC-W. WT cells were then used to demarcate unstained population using the marker gate consequently gating for GFP+ population termed as P1 using FITC-A fluorescence plotted on the x-axis and count on the y-axis.

☒ Tick this box to confirm that a figure exemplifying the gating strategy is provided in the Supplementary Information.
